# Novel perspectives on the rare hemophagocytic lymphohistiocytosis: insights from a multi-center retrospective cohort

**DOI:** 10.3389/fmed.2026.1809705

**Published:** 2026-04-15

**Authors:** Changkun Chen, Yanquan Liu, Xiaojun Chen, Hehui Zhang, Jianzhen Shen, Zuotao Li, Yue Yin, He Huang

**Affiliations:** 1 Department of Hematology, Ganzhou People’s Hospital (The Affiliated Ganzhou Hospital of Nanchang University), Jiangxi Health Commission Key Laboratory of Leukemia, Ganzhou, Jiangxi, China; 2 Department of Hematology, The Affiliated Hospital of Putian University, Putian, Fujian, China; 3 Department of Intensive Medicine (Comprehensive ICU), The First Affiliated Hospital of Gannan Medical University, Ganzhou, Jiangxi, China; 4 Department of Hematology, Fujian Medical University Union Hospital, Fuzhou, Fujian, China; 5 Department of Cardiovascular Medicine, The First Affiliated Hospital of Gannan Medical University, Ganzhou, Jiangxi, China

**Keywords:** clinical features, hemophagocytic lymphohistiocytosis, malignant hematological diseases, prognostic analysis, risk factors

## Abstract

**Background and objective:**

Hemophagocytic syndrome, also known as hemophagocytic lymphohistiocytosis (HLH), is a rare clinical disease that is highly challenging to diagnose, has a low long-term survival rate and a high mortality rate. Currently, due to the particularity of HLH and the lack of effective understanding of the severity and prognosis of the disease in clinical practice, HLH patients are often missed or misdiagnosed in clinical practice, causing them to miss the best opportunity for diagnosis and treatment. This study aimed to retrospectively summarize and analyze the clinical characteristics, diagnosis and treatment, and prognosis of HLH patients from four medical centers, aiming to explore the risk factors affecting the prognosis of HLH patients and further enhance the understanding of HLH.

**Methods:**

The clinical data of 162 patients with HLH diagnosed in four medical centers from May 2017 to May 2025 were collected. The general conditions, laboratory results, diagnosis and treatment processes, and prognosis of these patients were analyzed, and univariate and multivariate analyses of prognostic factors were conducted.

**Results:**

A total of 162 HLH patients were included in this study, of whom 90 cases survived (55.56%), 72 cases died (44.44%), and there were 78 male patients (48.15%) and 84 female patients (51.85%), with a median age at onset of 52 years (range: 1–83 years). The most common etiological factor was Epstein–Barr virus (EBV) infection, and the primary presenting symptom was fever. First-line treatment primarily involved anti-infective therapy and symptomatic management, which was administered to 147 cases (90.74%). Prognostic analysis revealed that age, history of malignancy, infection history, presence of dermatological symptoms, APTT, INR, PCT, CRP, TG, TBIL, ferritin level, sCD25, lactate, SOFA score and time to treatment initiation, glucocorticoid monotherapy, gamma globulin treatment were significantly associated with patient outcomes in HLH. Tumor history, ferritin level, sCD25, lactate, SOFA score, time to treatment initiation, gamma globulin treatment were independent risk factors for mortality. Univariate Cox regression analysis identified that age, tumor history, history of rheumatic and autoimmune disorders, CRP, ferritin level, sCD25, lactate, SOFA score and time to treatment initiation, chemotherapy, glucocorticoid monotherapy, gamma globulin treatment were significant prognostic factors for OS in HLH patients. Multivariate analysis confirmed that tumor history, CRP, ferritin level, sCD25, lactate, SOFA score and time to treatment initiation, chemotherapy, glucocorticoid monotherapy, gamma globulin treatment were independent prognostic factors affecting OS in HLH patients. Age, APTT, INR, ferritin level, SOFA score, time to treatment initiation, glucocorticoid monotherapy, gamma globulin treatment were independent prognostic factors affecting OS in infection-triggered HLH patients. While age, INR, ferritin level, SOFA score, time to treatment initiation, chemotherapy were independent prognostic factors affecting OS in malignancy-associated HLH patients. Combined therapy regimens (especially chemotherapy combined with gamma globulin, glucocorticoid combined with gamma globulin) showed better clinical efficacy and survival benefits in the treatment of HLH.

**Conclusion:**

HLH is a rare clinical disease, EBV was the predominant trigger for HLH. Prognostic factors differed between infection- and malignancy-associated subgroups. Combined regimens, particularly those including gamma globulin, offered superior survival benefits, underscoring the need for etiology-specific treatment strategies.

## 1 Introduction

Hemophagocytic lymphohistiocytosis (HLH), also known as hemophagocytic syndrome, is a rare syndrome of high inflammatory response caused by abnormal activation of immune cells due to various potential pathogenic factors and secretion of a large amount of inflammatory cytokines (1). The main clinical manifestations include fever, hepatosplenomegaly, pancytopenia, and hemophagy in bone marrow, liver, spleen, and lymph node tissues, etc. (2, 3). According to the cause, HLH can be classified into primary HLH and secondary HLH (4). Primary HLH is caused by gene defects that lead to excessive activation of immune cells, and secondary HLH could be caused by various inducing factors such as infection, tumors, rheumatic immune diseases, and drugs (5, 6). A definite genetic defect is the gold standard for diagnosing primary HLH (7).

However, it is regrettable that the clinical manifestations of HLH patients are mainly non-specific and highly confusing symptoms or signs such as persistent fever, hepatosplenomegaly and pancytopenia, which undoubtedly pose a challenge to the early clinical diagnosis and treatment of HLH (8, 9). Therefore, this multicenter retrospective study collected and analyzed clinical data from HLH patients across four tertiary medical centers in different regions of China. By summarizing the clinical characteristics, diagnostic and therapeutic experiences, and prognostic factors, we aimed to provide a more comprehensive reference for the clinical practice and prognostic assessment of HLH, particularly highlighting potential regional similarities and differences in disease presentation and management.

## 2 Materials and methods

### 2.1 Research object

**Figure 1:**
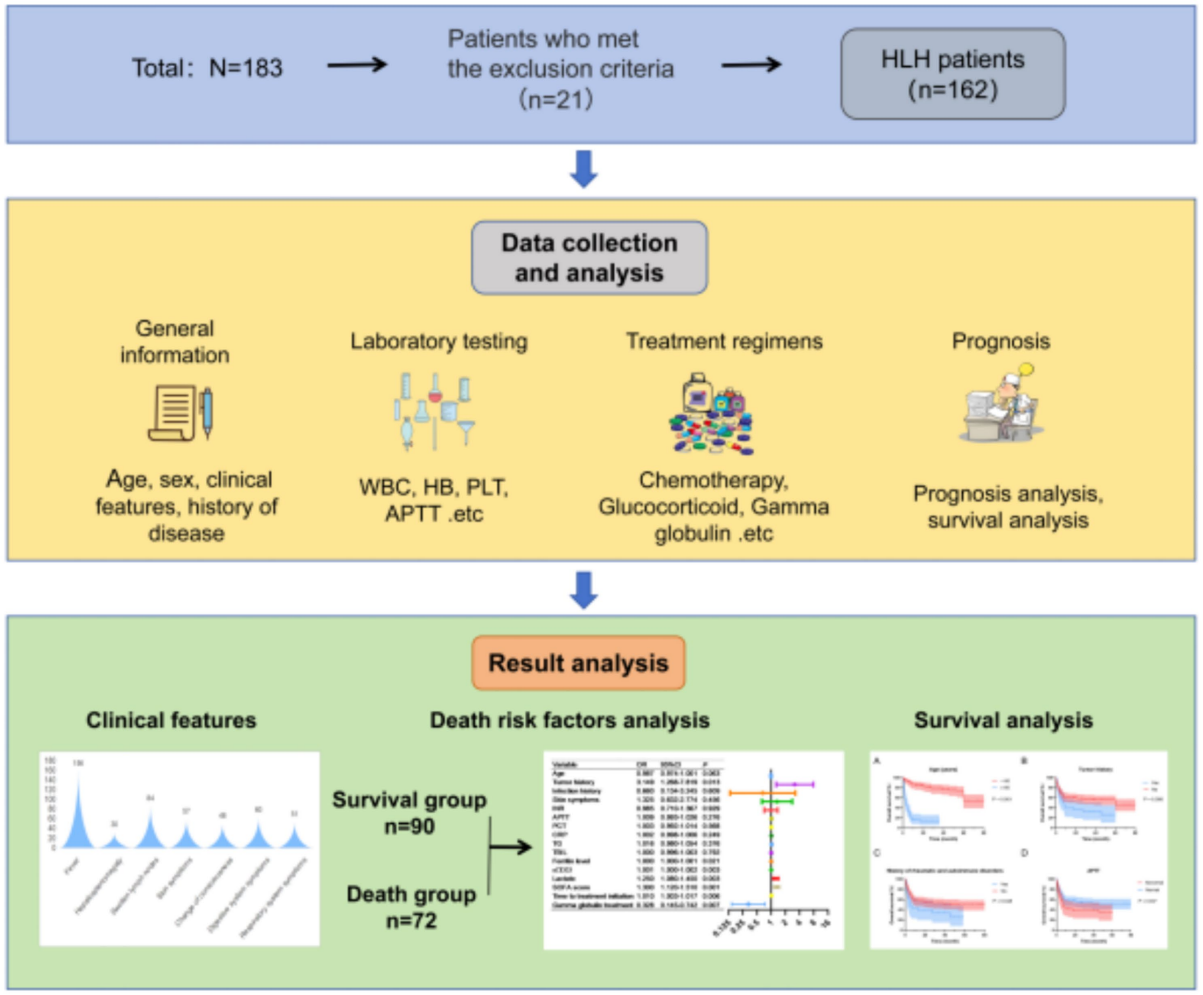
Technical research roadmap of this study.

This study included 162 patients with HLH who were diagnosed and treated at four medical centers, namely the Ganzhou People’s Hospital, the First Affiliated Hospital of Gannan Medical University, the Affiliated Hospital of Putian University and Fujian Medical University Union Hospital, from May 2017 to May 2025. This study has been approved by the medical ethics committees of each medical center and complies with the principles of the Declaration of Helsinki and relevant ethical requirements. No intervention measures were taken against patients during the research process, and the interests of patients were not harmed. An application for exemption from informed consent has been made and approved. The technical roadmap for this study is detailed in Figure 1.

### 2.2 Inclusion and exclusion criteria

Inclusion criteria for this study: (1) Meeting the diagnostic criteria of HLH-2004 (10), and having not received any clinical intervention in other hospitals; (2) Those with complete case data. Exclusion criteria: (1) Pregnant women, lactating women or those with plans to become pregnant in the near future; (2) Those who died within 48 h after enrollment.

### 2.3 Clinical data and information

This study collected clinical data of patients, including but not limited to the gender of all patients, their history of underlying diseases, as well as their age, etiology, Fer, TG, FIB, LDH, albumin (ALB), serum creatinine (Cr), ALT, HGB, PLT and lymphocyte count (LYR) and other blood biochemical indicators at the initial diagnosis of HLH. The presence of liver, spleen and superficial lymph node enlargement in the patient is determined through plain CT scan of the chest and abdomen and B-ultrasound.

### 2.4 Diagnostic criteria

The diagnosis of HLH refers to the HLH-2004 diagnostic criteria of the International Histiocytic Society (10). A diagnosis can be made if five of the following eight criteria are met: (1) Fever; (2) Enlarged spleen; (3) Cytopenia, involving two or three blood lines in the peripheral blood; (4) Hypertriglyceridemia and/or hypofibrinogenemia, fasting triglyceride (TG) ≥ 3.0 mmol/L, fibrinogen (FIB) ≤ 1.5 g/L; (5) Elevated serum ferritin (Fer) (≥500 μg/L); (6) Soluble interleukin-2 receptor (sCD25) ≥ 2,400 U/mL; (7) Reduced or absent NK cell activity; Bone marrow, spleen or lymph node biopsies show phagocytic blood cells, but there is no evidence of malignant tumor disease.

### 2.5 Morphological examination of hemophagocytic cells

All patients enrolled in this study underwent bone marrow aspiration to confirm the presence of hemophagocytosis. Bone marrow smears were prepared and stained using the Wright-Giemsa staining method. The detailed staining procedure is as follows: Prior to staining, the Wright-Giemsa staining solution was thoroughly mixed. Bone marrow smears from patients with HLH were allowed to air-dry completely. Two to four fresh, well-prepared bone marrow smears with adequate cellular content were selected. The Wright-Giemsa staining solution was then applied to fully cover the smear. The smear was fixed for 15–30 s, allowing the formaldehyde in the staining solution to preserve cellular morphology. Subsequently, an equal volume of phosphate buffer (pH 6.4) was added, and the slide was gently agitated and left to stain for 20 min. The excess stain was then rinsed off with gently flowing clean water. The slide was carefully blotted dry using sterile absorbent paper without rubbing or disturbing the smear. Once dry, the stained smear was examined under a microscope from multiple fields, including the top, bottom, left, right, and central regions, and representative images were captured immediately.

### 2.6 Therapeutic effect evaluation

Therapeutic effect evaluation criteria: referring to the therapeutic effect evaluation criteria formulated by the Midwestern Collaborative Group of the United States (11, 12), the following six indicators are evaluated: soluble CD25 (sCD25) level, ferritin level, blood cell count, triglyceride level, hemophagy phenomenon, consciousness status [in patients with central nervous system (CNS) involvement]. Complete response (CR): all the above indicators return to normal. Partial response (PR): ≥ 2 symptoms/laboratory indicators improve by more than 25%, and individual indicators also need to meet the following standards: (1) sCD25 decreases by more than 1/3; (2) Ferritin and triglycerides decrease by more than 25%; (3) In the absence of blood transfusion: For patients with neutrophil counts below 0.5 × 10^9^/L, the count must double and exceed 0.5 × 10^9^/L to be considered effective. For those with neutrophil counts ranging from 0.5 × 10^9^/L to 2.0 × 10^9^/L, normalization of the count is required. (4) For ALT levels exceeding 400 U/L, a reduction of more than 50% is necessary. Non-response (NR): Failure to meet the aforementioned criteria. Refractory HLH is defined as a failure to achieve remission or continued disease progression after 8 weeks of standardized treatment.

### 2.7 Prognosis follow-up

The inpatient treatment status of the patients was confirmed by reviewing their electronic and paper medical records. The patients were followed up by phone, and the follow-up period ended in June 2025. Overall survival (OS) is defined as the time interval from the diagnosis of HLH in a patient to death from any cause or the end of follow-up.

### 2.8 Statistical analysis

Statistical analysis was performed using SPSS 26.0 software. Normal distribution measurement data were expressed as Mean ± SD. Independent sample *t*-test was used for comparison between two groups, and one-way analysis of variance was used for comparison among three groups. Skewed distribution measurement data were expressed as *M* (*Q1*, *Q3*). The Mann–Whitney U test was used for comparison between two groups, and the Kruskal-Wallis H test was used for comparison among three groups. Counting data were expressed as the number of cases (%), and comparisons between groups were conducted using the χ^2^ test or Fisher’s exact probability method. The overall survival rate was analyzed using the Kaplan–Meier survival curve, and the log-rank test was used to compare the overall survival rates between groups. The analysis of prognostic influencing factors was conducted using the Cox proportional hazards regression model. The factors with *p* < 0.05 in the single-factor analysis were included in the multivariate regression analysis. Two-sided test, test level *α* = 0.05.

## 3 Results

### 3.1 General information of HLH patients

**Figure 2:**
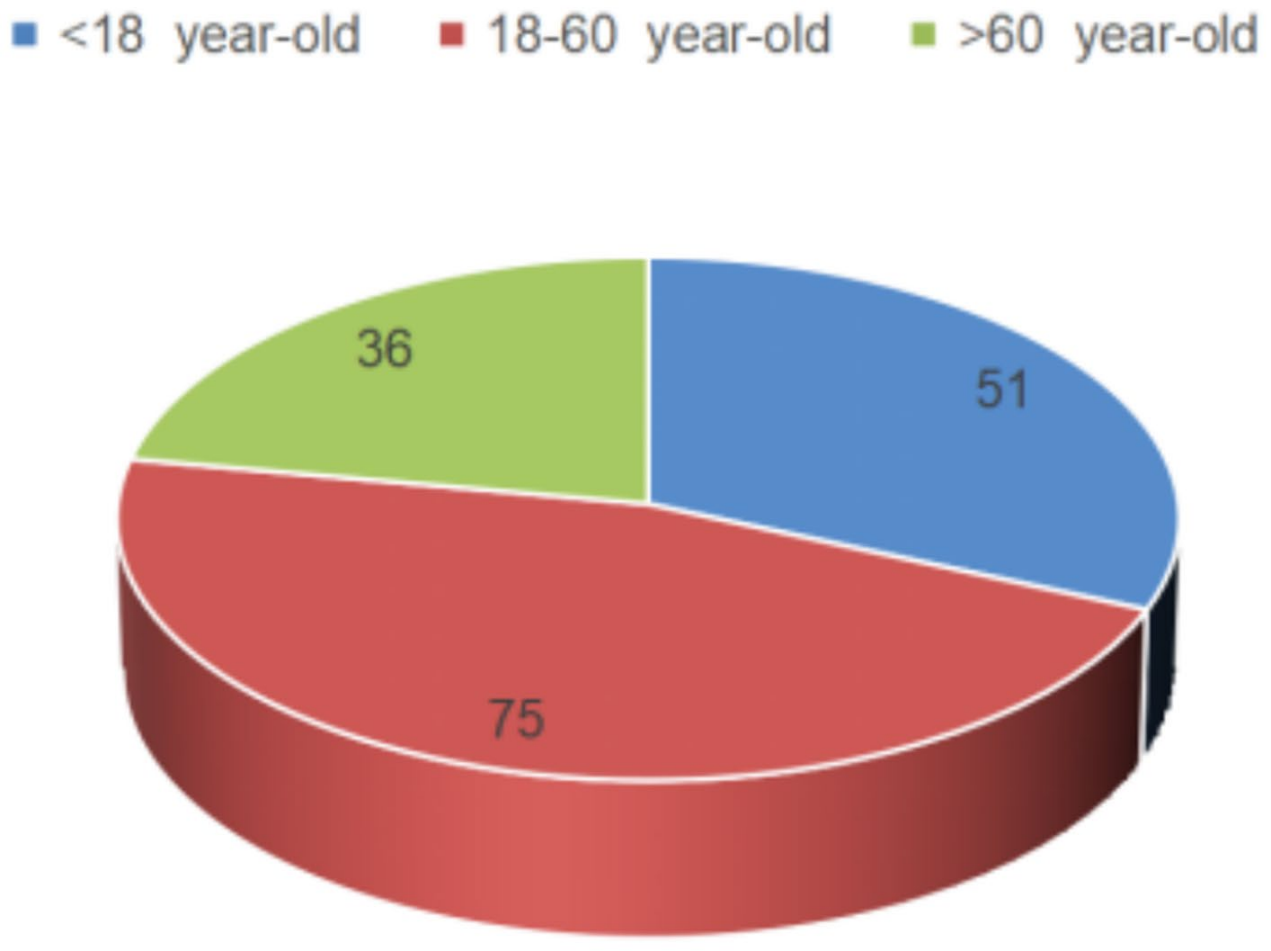
The distribution of 162 patients with HLH across different age groups.

**Figure 3:**
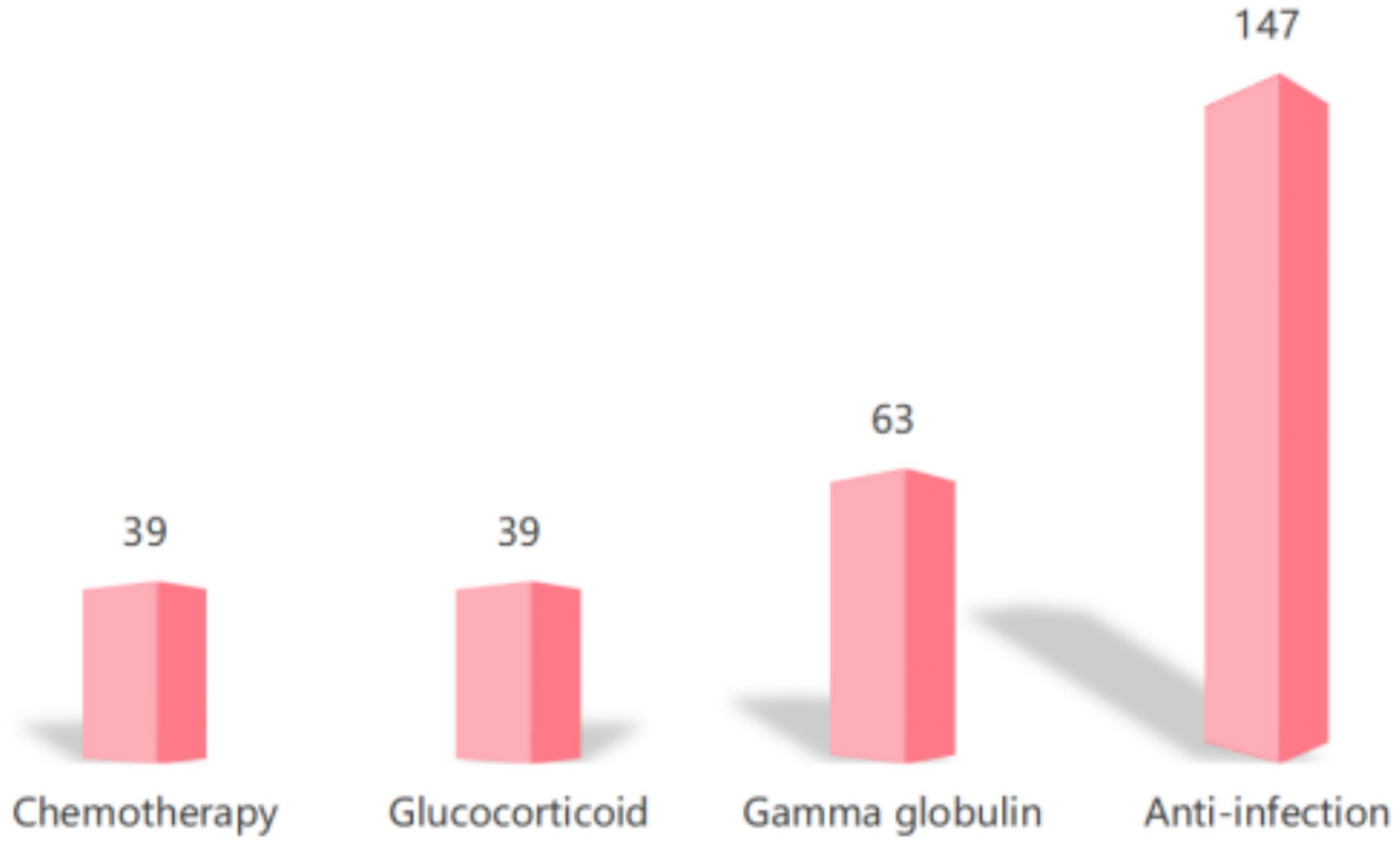
The main treatment methods and regimens for 162 patients with HLH.

According to the inclusion and exclusion criteria, combined with follow-up, a total of 162 cases of HLH patients were included in this study. Among them, 72 patients (44.44%) were transferred to the ICU for treatment, of which 90 cases survived (55.56%), 72 died (44.44%). There were 78 male cases (48.15%) and 84 female cases (51.85%), with a median age of onset of 52 years (ranging from 1 to 83 years). Interestingly, the age distribution of HLH patients included in this study was shown in Figure 2. The most common chief complaint is fever. Correspondingly, as depicted in Figure 3, the first-line treatment of HLH patients mainly focused on anti-infection and symptomatic treatment [147 cases (90.74%)]. Among them, 63 cases (38.89%) were treated with gamma globulin treatment, and only 39 cases (24.07%) received chemotherapy. Besides, 21 cases (12.96%) were treated with the HLH-1994 regimen, and 39 cases (24.07%) were treated with glucocorticoid monotherapy.

### 3.2 Distribution of underlying causes in patients with HLH

As shown in Table 1, among the 162 cases of HLH patients, 101 cases were infection-associated. Epstein–Barr virus (EBV) infection was the most common cause, accounting for 33.95%. Cytomegalovirus (CMV) infection accounted for 5 cases. Other infectious agents included influenza virus, respiratory syncytial virus, Coxsackievirus, HIV, *Mycobacterium tuberculosis*, *Streptococcus pneumoniae*, Staphylococcus species, *Acinetobacter baumannii*, and fungal pathogens. There were 49 cases of non-infectious HLH, including 33 cases (20.37%) associated with malignancies and 16 cases (9.88%) linked to rheumatologic or autoimmune disorders. The etiology remained undetermined in 12 cases (7.41%). Notably, some patients had multiple underlying conditions contributing to HLH. Specifically, 28 patients had concurrent malignant tumors and EBV infection, while 5 patients exhibited both autoimmune disease and EBV infection.

**Table 1:** Distribution of underlying causes in patients with HLH.

Underlying Causes	*n* (%)
Infectious agents	101 (62.35%)
Epstein–Barr virus (EBV)	55 (33.95%)
Cytomegalovirus (CMV)	5 (3.09%)
Influenza virus	5 (3.09%)
Respiratory syncytial virus	4 (2.47%)
Coxsackievirus	4 (2.47%)
Human immunodeficiency virus (HIV)	1 (0.62%)
*Mycobacterium tuberculosis*	5 (3.09%)
*Streptococcus pneumoniae*	4 (2.47%)
Staphylococcus	3 (1.85%)
*Acinetobacter baumannii*	3 (1.85%)
Fungi	2 (1.23%)
Mycoplasma	1 (0.62%)
Unknown pathogens	9 (5.56%)
Neoplastic conditions	33 (20.37%)
T-cell lymphoma	19 (11.73%)
B-cell lymphoma	9 (5.56%)
Leukemia	2 (1.23%)
Myelodysplastic syndrome	1 (0.62%)
Other solid tumors	2 (1.23%)
Rheumatologic and autoimmune diseases	16 (9.88%)
Adult-onset Still’s disease	5 (3.09%)
Dermatomyositis	3 (1.85%)
Sjögren’s syndrome	1 (0.62%)
Systemic lupus erythematosus	2 (1.23%)
Rheumatoid arthritis	2 (1.23%)
Undifferentiated connective tissue disease	3 (1.85%)
Cause of disease undetermined	12 (7.41%)

### 3.3 Clinical characteristics of patients with HLH

**Figure 4:**
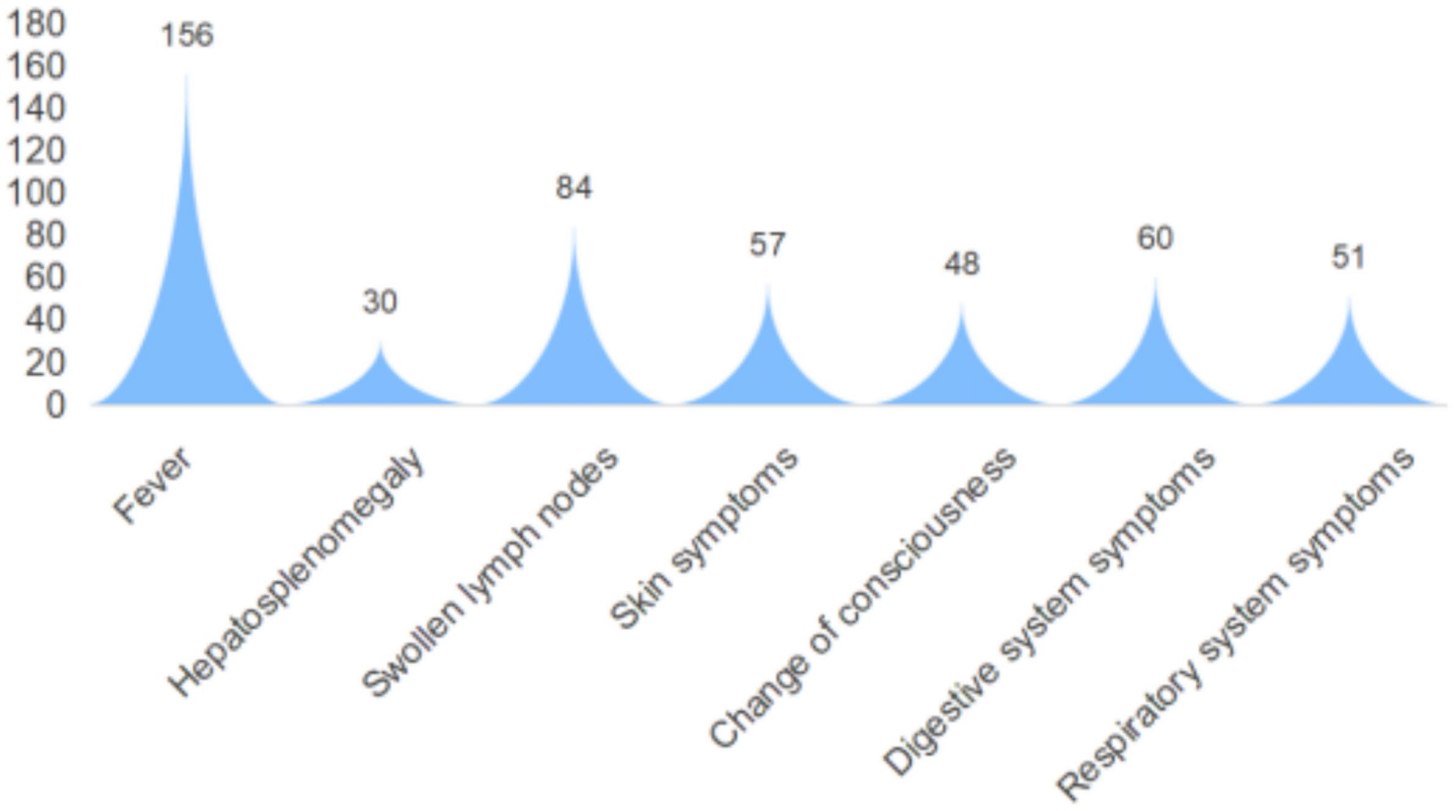
Clinical characteristics of 162 patients with HLH.

As illustrated in Figure 4, among the 162 cases of HLH patients analyzed, 156 cases presented with fever, representing the most common clinical manifestation. Hepatosplenomegaly and lymphadenopathy were observed in 30 (18.52%) and 84 (51.85%) patients, respectively. Skin manifestations and neurological symptoms were reported in 57 (35.19%) and 48 (29.63%) patients, respectively. Additionally, 60 cases (37.04%) and 51 cases (31.48%) of HLH patients experienced gastrointestinal and respiratory symptoms, respectively. Notably, 137 patients (84.57%) developed pneumonia during the course of their illness. Baseline clinical characteristics are summarized in Table 2. Based on follow-up outcomes, patients were categorized into survival and mortality groups. Statistically significant differences were observed between the two groups regarding age, history of malignancy, prior infections, and presence of dermatological symptoms. However, no significant differences were found between the groups in terms of gender, history of rheumatologic or immune disorders, presence of fever, lymphadenopathy, hepatosplenomegaly, altered mental status, or involvement of the gastrointestinal or respiratory systems.

**Table 2:** Baseline characteristics of general clinical data for patients with HLH.

Indexes		Outcome		χ^2^	*P*
		Survivors	Non-survivors		
Age (years)	< 60	63	57	12.164	**< 0.001**
	≥ 60	27	15		
Gender	male	42	36	0.178	0.673
	female	48	36		
History of malignancy	Yes	12	21	6.182	**0.013**
	No	78	51		
History of infection	Yes	47	34	9.951	**0.002**
	No	27	54		
History of rheumatic and autoimmune disorders	Yes	5	11	3.600	0.058
	No	82	64		
Lymphadenopathy	Yes	50	34	1.113	0.292
	No	40	38		
Hepatosplenomegaly	Yes	15	15	0.460	0.498
	No	75	57		
Dermatological symptoms	Yes	25	32	4.872	**0.027**
	No	65	40		
Altered mental status	Yes	28	20	0.003	0.959
	No	66	48		
Gastrointestinal symptoms	Yes	30	30	1.191	0.275
	No	60	42		
Respiratory symptoms	Yes	28	23	0.013	0.910
	No	62	49		

The bold values indicate statistically significant differences (*p* < 0.05) between groups or statistically significant prognostic factors identified in the analysis, as specified in the statistical methods section of the article.

### 3.4 Laboratory findings in HLH patients

Among the 162 patients diagnosed with HLH, over 90% exhibited abnormalities in hemoglobin levels, neutrophil counts, D-dimer, CRP, LDH, NK cell activity, ferritin, and sCD25 levels. Only 19 patients (11.73%) had serum ferritin levels below 1,000 μg/L, whereas 85 patients (52.47%) had ferritin levels exceeding 2000 μg/L. Detailed laboratory parameters are summarized in Table 3.

**Table 3:** Laboratory test indicators for 162 patients with HLH.

Test items	Indicator situation	*n* (%)
WBC	Normal	42 (25.93%)
	Abnormal	120 (74.07%)
HB	Normal	15 (9.26%)
	Abnormal	147 (90.74%)
PLT	Normal	54 (33.33%)
	Abnormal	108 (66.67%)
Neutrophil count	Normal	3 (1.85%)
	Abnormal	159 (98.15%)
PT	Normal	24 (14.81%)
	Abnormal	138 (85.19%)
APTT	Normal	45 (27.78%)
	Abnormal	117 (72.22%)
INR	Normal	99 (61.11%)
	Abnormal	63 (38.89%)
FIB	Normal	51 (31.48%)
	Abnormal	111 (68.52%)
D-dimer level	Normal	3 (1.85%)
	Abnormal	159 (98.15%)
PCT	Normal	33 (20.37%)
	Abnormal	129 (79.63%)
CRP	Normal	15 (9.26%)
	Abnormal	147 (90.74%)
IL-6	Normal	21 (12.96%)
	Abnormal	141 (87.04%)
ALT	Normal	39 (24.07%)
	Abnormal	123 (75.93%)
AST	Normal	24 (14.81%)
	Abnormal	138 (85.19%)
TG	Normal	60 (37.04%)
	Abnormal	102 (62.96%)
ALB	Normal	42 (25.93%)
	Abnormal	120 (74.07%)
LDH	Normal	9 (5.56%)
	Abnormal	153 (94.44%)
TBIL	Normal	63 (38.89%)
	Abnormal	99 (61.11%)
Natural Killer (NK) cell activity	Normal	3 (1.85%)
	Abnormal	159 (98.15%)
Ferritin level	Normal	4 (2.47%)
	Abnormal	158 (97.53%)
sCD25	Normal	12 (7.41%)
	Abnormal	150 (92.59%)
Lactate level	<1.6	95 (58.64%)
	≥1.6	67 (41.36%)

### 3.5 Morphological characteristics of hemophagocytic cells

**Figure 5:**
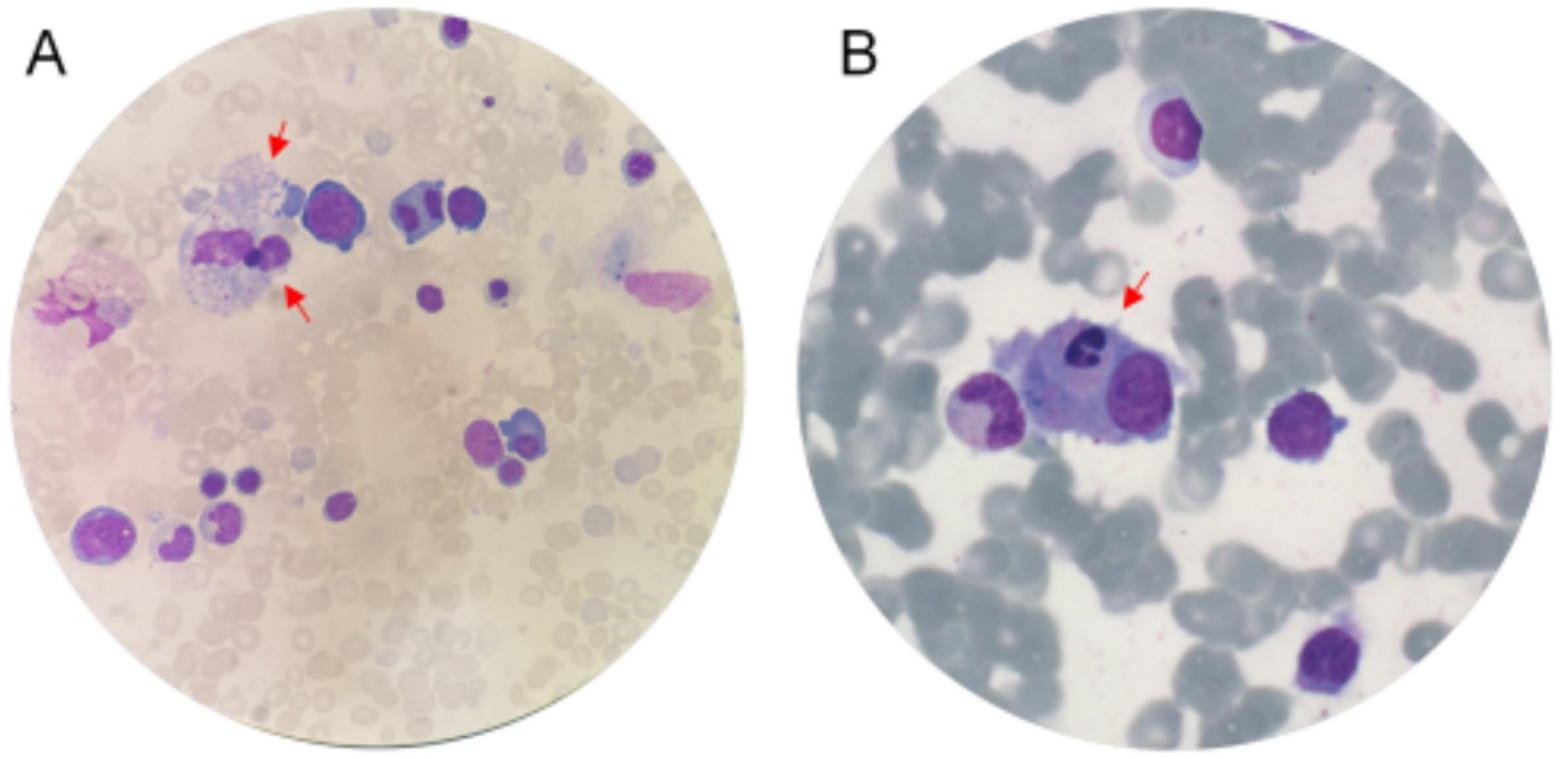
Morphological characteristics of HLH. **(A)** The red arrow indicates that phagocytosis of platelets, white blood cells, and erythroblasts can be observed. **(B)** The phenomenon of phagogranulocytes can be seen as indicated by the red arrow.

As illustrated in Figure 5, hemophagocytic cells are typically large in size, with abundant cytoplasm and irregular cell borders, which may appear tear-shaped or exhibit pseudopodia. The cytoplasmic staining characteristics are variable, appearing reddish, light gray, turbid, or with a frosted-glass appearance. The nucleus is generally medium-sized and may be oval, horseshoe-shaped, kidney-shaped, or irregular in form, often located eccentrically. Nuclear chromatin appears loosely arranged and reticular, with one or two nucleoli occasionally visible. A single hemophagocytic cell may contain varying numbers of intact nucleated red blood cells, mature red blood cells, white blood cells, platelets, or cellular debris, with red blood cells and platelets being the most commonly observed ingested components. Some cells may also exhibit cytoplasmic granules and multiple vacuoles, with indistinct cytoplasmic boundaries.

### 3.6 Efficacy comparison of different treatment regimens in HLH patients

The first-line treatment predominantly consisted of anti-infective and supportive therapies (*n* = 147, 90.74%). Among these, 63 patients (38.89%) received intravenous gamma globulin treatment, while only 39 patients (24.07%) underwent chemotherapy. Of those receiving chemotherapy, 21 (12.96%) were treated according to the HLH-1994 protocol, and 39 cases (24.07%) received corticosteroid monotherapy. Nine patients (5.55%) received concurrent chemotherapy and gamma globulin treatment therapy. Additionally, 12 patients (7.41%) were treated with both corticosteroids and gamma globulin treatment, and 5 patients (3.09%) received combined chemotherapy and steroid therapy. Based on the therapeutic effect evaluation criteria, the clinical efficacy of different treatment regimens was statistically analyzed, with the results of CR, PR and NR rates compared by the chi-square test. The overall response rate (ORR) was defined as the sum of CR and PR rates. As shown in Table 4, significant differences were observed in the distribution of therapeutic efficacy among different treatment regimens (χ^2^ = 52.689, *p* < 0.001). The combined therapy of chemotherapy and gamma globulin had the highest CR rate (44.44%) and ORR (77.78%), followed by the combination of glucocorticoids and gamma globulin (CR: 33.33%, ORR: 66.67%) and the HLH-1994 regimen (CR: 28.57%, ORR: 61.90%). VP-16 therapy alone showed moderate therapeutic efficacy (CR: 23.53%, ORR: 58.82%). In contrast, anti-infective and supportive therapy alone had the lowest CR rate (8.16%) and ORR (34.01%), with an NR rate as high as 65.99%. The CR rate and ORR of glucocorticoid monotherapy (15.38, 46.15%) and chemotherapy alone (17.95, 48.72%) were at a moderate level, with no significant statistical difference between the two regimens (*p* > 0.05).

**Table 4:** Efficacy comparison of different treatment regimens for HLH patients.

Treatment regimen	*n*	CR	PR	NR	ORR (CR + PR)
Anti-infective and supportive therapy	147	12(8.16%)	38(25.85%)	97(65.99%)	50(34.01%)
Glucocorticoid monotherapy	39	6(15.38%)	12(30.77%)	21(53.85%)	18(46.15%)
Chemotherapy alone	39	7(17.95%)	12(30.77%)	20(51.28%)	19(48.72%)
VP-16 monotherapy	17	4(23.53%)	6(35.29%)	7(41.18%)	10(58.82%)
HLH-1994 treatment	21	6(28.57%)	7(33.33%)	8(38.10%)	13(61.90%)
Glucocorticoid + gamma globulin	12	4(33.33%)	4(33.33%)	4(33.33%)	8(66.67%)
Chemotherapy + gamma globulin	9	4(44.44%)	3(33.33%)	2(22.22%)	7(77.78%)
Chemotherapy + glucocorticoid	5	1(20.00%)	2(40.00%)	2(40.00%)	3(60.00%)
Total	162	44(27.16%)	84(51.85%)	34(20.99%)	128(79.01%)
***χ*** ^**2**^	–	52.689	–	–	–
*P*	–	**<0.001**	–	–	–

### 3.7 Risk factor analysis for mortality

**Figure 6:**
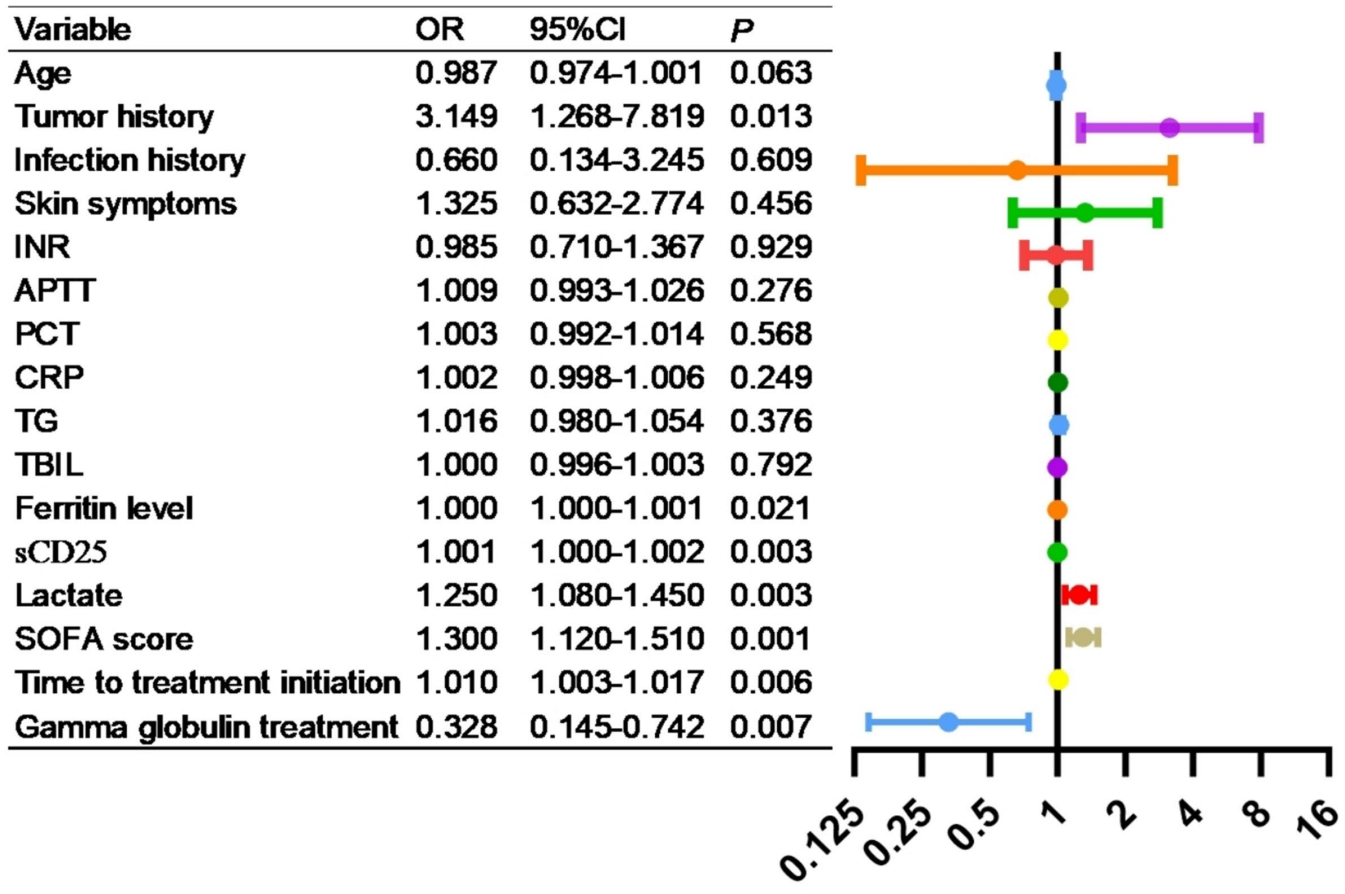
Multivariate logistic regression analysis of risk factors for mortality in HLH patients.

As shown in Table 5, factors influencing prognosis were analyzed, and variables including age, history of malignancy, infection history, presence of dermatological symptoms, APTT, INR, PCT, CRP, TG, TBIL, ferritin level, sCD25, lactate level, SOFA score and time to treatment initiation, glucocorticoid alone treatment, gamma globulin treatment were found to be significantly correlated with patient outcomes (*p* < 0.05). As shown in Figure 6, factors with *p* < 0.05 in the univariate analysis were included in multivariate analysis, revealing that tumor history, ferritin level, sCD25, lactate level, SOFA score, time to treatment initiation, gamma globulin treatment were independent risk factors for mortality.

**Table 5:** Univariate analysis of risk factors for mortality in HLH patients.

Parameters	Variables/groupings	Survival group *n* (%)	Death group *n* (%)	*Z/χ* ^2^	*P*
		*n* = 90	*n* = 72		
Age (years)	< 60	63 (70.00%)	57(79.17%)	12.164	<0.001
	≥ 60	27 (30.00%)	15(20.83%)		
Gender	Male	42 (46.67%)	36 (50.00%)	0.178	0.673
	Female	48 (53.33%)	36 (50.00%)		
Tumor history	Yes	12 (13.33%)	21 (29.17%)	6.182	**0.013**
	No	78 (86.67%)	51 (70.83%)		
Infection history	Yes	34 (37.78%)	54 (75.00%)	22.335	**<0.001**
	No	56 (62.22%)	18 (25.00%)		
History of rheumatic and autoimmune disorders	Yes	5 (5.56%)	11 (15.28%)	3.600	0.058
	No	82 (91.11%)	64 (88.89%)		
Lymphadenopathy	Yes	50 (55.56%)	34 (47.22%)	1.113	0.292
	No	40 (44.44%)	38 (52.78%)		
Hepatosplenomegaly	Yes	15 (16.67%)	15 (20.83%)	0.460	0.498
	No	75 (83.33%)	57 (79.17%)		
Dermatological symptoms	Yes	25 (27.78%)	32 (44.44%)	4.872	**0.027**
	No	65 (72.22%)	40 (55.56%)		
Altered mental status	Yes	28 (31.11%)	20 (27.78%)	0.003	0.959
	No	66 (73.33%)	48 (66.67%)		
Gastrointestinal symptoms	Yes	30 (33.33%)	30 (41.67%)	1.191	0.275
	No	60 (66.67%)	42 (62.69%)		
Respiratory symptoms	Yes	28 (31.11%)	23 (31.94%)	0.013	0.910
	No	62 (68.89%)	49 (68.05%)		
WBC	3.03 [0.16–17.29]	3.38 [0.68–18.89]	2.9 [0.16–24.89]	3.892	0.134
HB	90 [45–122]	117 [59–134]	78 [39–113]	0.227	0.657
PLT	77 [12–358]	101 [59–423]	38 [7–258]	0.149	0.728
Neutrophil count	57.2 [4.4–93]	69.9 [17.4–98.6]	21.45 [4.4–39.8]	0.356	0.917
PT	14.9 [9.1–44.3]	16 [13.3–25.4]	17 [12.5–28.4]	2.565	0.867
APTT	37.6 [19.7–170]	34.1 [28.8–52]	45.1 [30.6–58.1]	5.232	**<0.001**
INR	1.29 [1.03–1.76]	1.24 [1.04–1.81]	1.47 [1.06–2.37]	4.138	**<0.001**
FIB	1.94 [0.79–3.11]	2.07 [1.06–3.17]	1.73 [0.74–3.38]	8.463	0.562
D-dimer level	6.94 [2.17–22.27]	6.36 [2.17–21.33]	9.05 [2.28–25.04]	9.342	0.875
PCT	4.19 [0.91–23.06]	1.17 [0.23–19.02]	5.1 [1.04–40.4]	6.784	**<0.001**
CRP	76.21 [25.12–194.4]	46.46 [9.09–108.64]	96.99 [25.71–198.61]	6.413	**0.001**
IL-6	29.33 [7.31–96.89]	14.98 [7.31–76.67]	37.91 [17.09–395.1]	3.007	0.051
ALT	61 [37–144]	57 [37–126]	86 [41–210]	5.347	0.623
AST	146 [68–370]	113 [66–333]	164 [86–531]	0.998	0.834
TG	5.48 [2.31–11.31]	4.93 [2.31–10.85]	6.05 [2.39–12.22]	8.941	**0.001**
ALB	29.8 [23.9–32.6]	30.4 [24.8–34.2]	26.2 [18.2–31.6]	6.355	0.342
LDH	918 [520–3,183]	460 [317–891]	1,107 [764–6,442]	3.750	0.123
TBIL	23.8 [6.8–99.9]	13.9 [6.6–32.7]	32.7 [13.8–133.7]	8.064	**<0.001**
NK cell activity	5.14 [2.32–12.56]	5.99 [3.12–17.71]	3.15 [1.89–8.34]	5.609	0.211
Ferritin level	7,324 [1237–8,112]	5,086 [1140–7,086]	7,980 [3384–12,897]	2.520	**0.011**
sCD25	2,730 [1985–3,976]	2,385 [1834–2,697]	4,867 [3446–7,566]	4.986	**<0.001**
Lactate (mmol/L)	3.7 [1.1–8.6]	2.1 [0.8–5.3]	4.8 [1.5–9.7]	12.215	**<0.001**
SOFA score (points)	4.0 [2.0–9.0]	3.0 [1.0–6.0]	7.0 [3.0–11.0]	8.963	**<0.001**
VP-16 monotherapy	Yes	5 (5.56%)	4 (5.56%)	0.900	0.343
	No	3 (3.33%)	6 (8.33%)		
HLH-94 treatment	Yes	5 (5.56%)	7 (9.72%)	0.016	0.899
	No	4 (4.44%)	5 (6.94%)		
Time to treatment initiation (hours)	58.0 [24.0–108.0]	48.0 [24.0–72.0]	72.0 [36.0–120.0]	6.892	**<0.001**
Chemotherapy alone	Yes	18 (46.15%)	21 (53.85%)	0.774	0.379
	No	72 (58.54%)	51 (41.46%)		
Glucocorticoid monotherapy	Yes	25 (64.10%)	14 (35.90%)	4.578	**0.032**
	No	65 (52.85%)	58 (47.15%)		
Gamma globulin treatment	Yes	42 (62.69%)	25 (37.31%)	5.876	**0.015**
	No	48 (50.53%)	47 (49.47%)		

The bold values indicate statistically significant differences (*p* < 0.05) between groups or statistically significant prognostic factors identified in the analysis, as specified in the statistical methods section of the article.

### 3.8 Survival analysis of HLH patients

**Figure 7:**
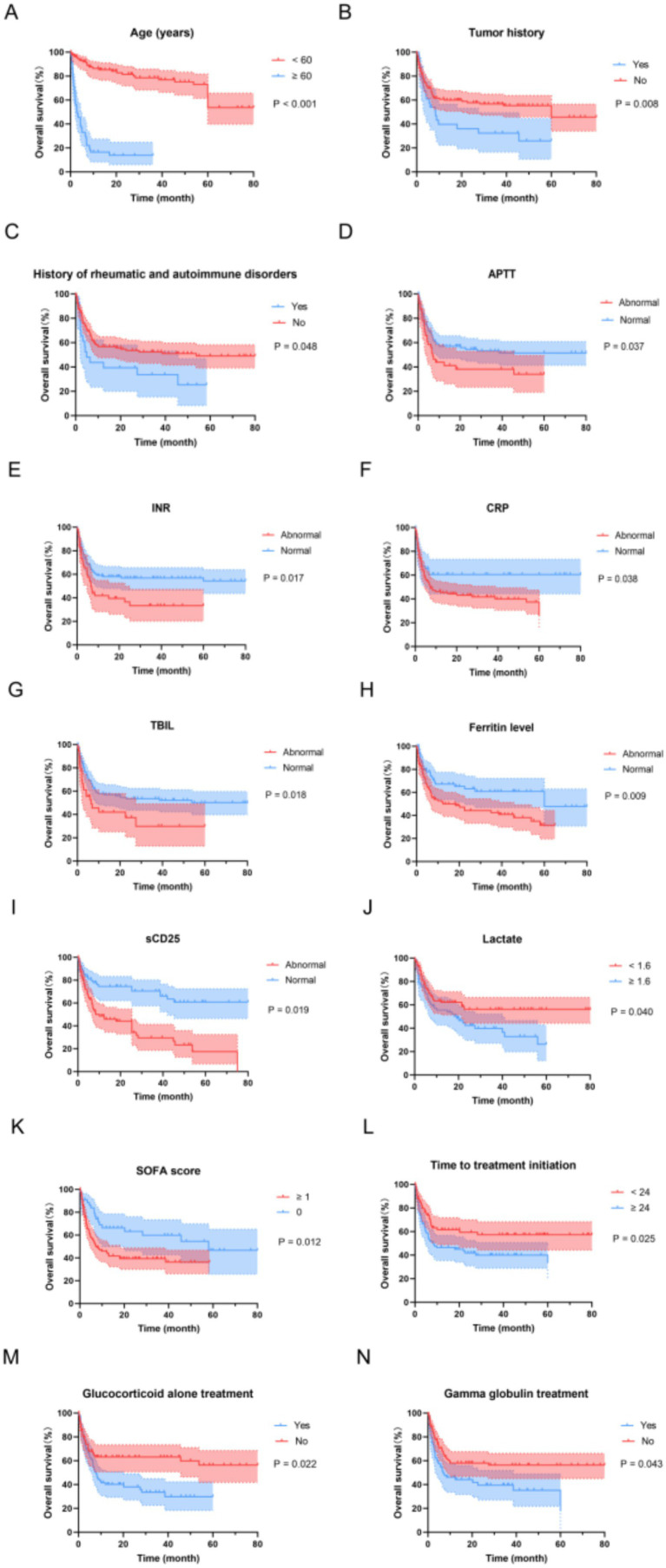
Kaplan–Meier overall survival curves for HLH patients. **(A)** The overall survival curves of age of HLH patients. **(B)** The overall survival curves of tumor history of HLH patients. **(C)** The overall survival curves of history of rheumatology and immunology of HLH patients. **(D)** The overall survival curves of APTT of HLH patients. **(E)** The overall survival curves of INR of HLH patients. **(F)** The overall survival curves of CRP of HLH patients. **(G)** The overall survival curves of TBIL of HLH patients. **(H)** The overall survival curves of Ferritin level of HLH patients. **(I)** The overall survival curves of sCD25 of HLH patients. **(J)** The overall survival curves of lactate of HLH patients. **(K)** The overall survival curves of SOFA score of HLH patients. **(L)** The overall survival curves of time to treatment initiation of HLH patients. **(M)** The overall survival curves of glucocorticoid monotherapy of HLH patients. **(N)** The overall survival curves of gamma globulin treatment of HLH patients.

Regarding OS, as of June 1, 2025, the follow-up duration ranged from 2 to 84 months, with a median survival time of 7.5 months. The 1-year survival rate was 68.27%, the 2-year survival rate was 58.32%, and the 3-year survival rate was 52.45%. In order to investigate the effects of various influencing factors on the survival of HLH patients, log-rank test was used for each indicator, and the results are shown in Table 6, The results showed that age, tumor history, history of rheumatic and autoimmune disorders, APTT, INR, CRP, TBIL, ferritin level, sCD25, lactate, SOFA score, time to treatment initiation, glucocorticoid alone treatment, gamma globulin treatment had influence on OS of HLH patients. The survival curve was drawn by Kaplan–Meier method, and the meaningful results were shown in Figure 7. Given the prognostic differences between malignancy-associated HLH and infection-associated HLH, univariate analyses of OS were conducted separately for each subgroup. The log-rank test was employed to evaluate the prognostic factors, and the results are presented in Table 6, and the results showed that age, infection history APTT, INR, CRP, Ferritin level, lactate, SOFA score, time to treatment initiation, glucocorticoid alone treatment, gamma globulin treatment had influence on OS of infection-triggered HLH patients (Table 7). Age, APTT, INR, CRP, TBIL, Ferritin level, Lactate, SOFA score, time to treatment initiation, chemotherapy had influence on OS of malignancy-associated HLH patients (Table 8).

**Table 6:** Clinical features of HLH patients and OS univariate analysis.

Parameters	Variables/groupings	Median survival period (months)	χ^2^	*P*
Age (years)	<60	15.0	11.320	**<0.001**
	≥60	3.0		
Gender	Male	7.0	0.169	0.681
	Female	8.0		
Tumor history	Yes	1.0	7.040	**0.008**
	No	18.0		
Infection history	Yes	4.0	2.620	0.106
	No	8.0		
History of rheumatic and autoimmune disorders	Yes	1.0	3.908	**0.048**
	No	12.0		
Lymphadenopathy	Yes	6.0	0.819	0.365
	No	8.0		
Hepatosplenomegaly	Yes	7.0	1.180	0.277
	No	8.0		
Dermatological symptoms	Yes	6.0	0.268	0.425
	No	8.0		
Altered mental status	Yes	7.0	0.052	0.819
	No	7.5		
Gastrointestinal symptoms	Yes	7.0	0.903	0.342
	No	8.0		
Respiratory symptoms	Yes	7.0	0.003	0.957
	No	7.5		
WBC	Normal	8.0	0.587	0.443
	Abnormal	7.0		
HB	Normal	8.0	0.081	0.775
	Abnormal	7.0		
PLT	Normal	9.0	3.555	0.059
	Abnormal	7.0		
Neutrophil count	Normal	8.0	1.378	0.240
	Abnormal	7.0		
PT	Normal	8.0	2.962	0.085
	Abnormal	6.0		
APTT	Normal	10.0	4.356	**0.037**
	Abnormal	3.0		
INR	Normal	9.0	5.651	**0.017**
	Abnormal	5.0		
FIB	Normal	8.0	0.290	0.590
	Abnormal	7.0		
D-dimer level	Normal	8.0	0.114	0.736
	Abnormal	7.0		
PCT	Normal	8.5	1.174	0.279
	Abnormal	7.5		
CRP	Normal	10.0	4.301	**0.038**
	Abnormal	6.0		
IL-6	Normal	8.0	0.777	0.378
	Abnormal	7.0		
ALT	Normal	8.0	1.061	0.303
	Abnormal	7.0		
AST	Normal	8.0	3.441	0.064
	Abnormal	6.0		
TG	Normal	8.0	0.144	0.705
	Abnormal	7.0		
ALB	Normal	8.0	0.345	0.557
	Abnormal	7.0		
LDH	Normal	8.0	0.283	0.595
	Abnormal	7.0		
TBIL	Normal	9.0	5.635	**0.018**
	Abnormal	4.0		
NK cell activity	Normal	9.0	3.002	0.083
	Abnormal	5.0		
Ferritin level	Normal	11.0	10.99	0.009
	Abnormal	5.0		
sCD25	Normal	10.0	3.800	0.019
	Abnormal	6.0		
Lactate (mmol/L)	<1.6	9.0	4.213	0.040
	≥1.6	6.0		
SOFA score (points)	0	10.0	6.327	0.012
	≥1	5.0		
Time to treatment initiation (hours)	<24	9.0	5.841	0.025
	≥24	5.0		
VP-16 monotherapy	Yes	9.0	3.108	0.078
	No	7.0		
HLH-94 treatment	Yes	8.0	0.113	0.737
	No	7.0		
Chemotherapy	Yes	9.0	3.652	0.053
	No	4.0		
Glucocorticoid monotherapy	Yes	10.0	5.238	**0.022**
	No	6.0		
Gamma globulin treatment	Yes	9.5	4.105	**0.043**
	No	6.0		

The bold values indicate statistically significant differences (*p* < 0.05) between groups or statistically significant prognostic factors identified in the analysis, as specified in the statistical methods section of the article.

**Table 7:** Clinical features of infection-triggered HLH patients and OS univariate analysis.

Parameters	Variables/groupings	Median survival period (months)	χ^2^	*P*
Age (years)	<60	18.0	9.852	**<0.001**
	≥60	4.0		
Gender	Male	7.5	0.105	0.746
	Female	8.0		
Tumor history	Yes	6.0	0.321	0.571
	No	7.5		
Infection history	Yes	7.0	5.018	**0.025**
	No	7.5		
History of rheumatic and autoimmune disorders	Yes	6.5	0.156	0.693
	No	7.5		
Lymphadenopathy	Yes	7.0	0.628	0.428
	No	8.0		
Hepatosplenomegaly	Yes	7.5	0.915	0.339
	No	8.0		
Dermatological symptoms	Yes	6.5	0.214	0.643
	No	8.0		
Altered mental status	Yes	7.0	0.036	0.849
	No	7.5		
Gastrointestinal symptoms	Yes	7.0	0.782	0.377
	No	8.0		
Respiratory symptoms	Yes	7.0	0.002	0.965
	No	7.5		
WBC	Normal	8.5	0.496	0.481
	Abnormal	7.0		
HB	Normal	8.0	0.068	0.794
	Abnormal	7.5		
PLT	Normal	9.5	3.215	0.073
	Abnormal	7.0		
Neutrophil count	Normal	8.0	1.125	0.289
	Abnormal	7.0		
PT	Normal	8.0	2.658	0.103
	Abnormal	6.5		
APTT	Normal	10.5	3.986	**0.046**
	Abnormal	3.5		
INR	Normal	9.5	5.128	**0.024**
	Abnormal	5.5		
FIB	Normal	8.0	0.256	0.613
	Abnormal	7.5		
D-dimer level	Normal	8.0	0.098	0.754
	Abnormal	7.5		
PCT	Normal	8.5	1.015	0.314
	Abnormal	7.5		
CRP	Normal	10.0	3.895	**0.048**
	Abnormal	6.5		
IL-6	Normal	8.0	0.698	0.403
	Abnormal	7.5		
ALT	Normal	8.0	0.956	0.328
	Abnormal	7.5		
AST	Normal	8.0	3.102	0.078
	Abnormal	6.5		
TG	Normal	8.0	0.121	0.728
	Abnormal	7.5		
ALB	Normal	8.0	0.302	0.582
	Abnormal	7.5		
LDH	Normal	8.0	0.245	0.621
	Abnormal	7.5		
TBIL	Normal	9.5	0.089	0.765
	Abnormal	4.5		
NK cell activity	Normal	9.0	2.856	0.091
	Abnormal	5.5		
Ferritin level	Normal	11.5	9.865	**0.002**
	Abnormal	5.5		
sCD25	Normal	10.5	3.528	0.060
	Abnormal	6.5		
Lactate (mmol/L)	<1.6	9.5	3.965	**0.047**
	≥1.6	6.5		
SOFA score (points)	0	10.5	5.986	**0.014**
	≥1	5.5		
Time to treatment initiation (hours)	<24	9.5	5.256	**0.022**
	≥24	5.5		
VP-16 monotherapy	Yes	9.5	2.895	0.089
	No	7.5		
HLH-94 treatment	Yes	8.0	0.095	0.758
	No	7.5		
Chemotherapy	Yes	6.5	2.985	0.084
	No	8.0		
Glucocorticoid monotherapy	Yes	10.5	6.328	**0.012**
	No	6.5		
Gamma globulin treatment	Yes	10.0	5.782	**0.016**
	No	6.0		

The bold values indicate statistically significant differences (*p* < 0.05) between groups or statistically significant prognostic factors identified in the analysis, as specified in the statistical methods section of the article.

**Table 8:** Clinical features of malignancy-associated HLH patients and OS univariate analysis.

Parameters	Variables/groupings	Median survival period (months)	χ^2^	*P*
Age (years)	<60	3.0	8.965	**<0.001**
	≥60	1.0		
Gender	Male	1.0	0.189	0.664
	Female	1.5		
Tumor history	Yes	1.0	0.356	0.551
	No	1.5		
Infection history	Yes	1.0	0.098	0.754
	No	1.5		
History of rheumatic and autoimmune disorders	Yes	1.0	0.168	0.682
	No	1.5		
Lymphadenopathy	Yes	1.0	0.752	0.386
	No	2.0		
Hepatosplenomegaly	Yes	1.0	0.896	0.344
	No	2.0		
Dermatological symptoms	Yes	1.0	0.245	0.621
	No	2.0		
Altered mental status	Yes	1.0	0.048	0.826
	No	1.5		
Gastrointestinal symptoms	Yes	1.0	0.856	0.355
	No	2.0		
Respiratory symptoms	Yes	1.0	0.005	0.943
	No	1.5		
WBC	Normal	2.0	0.528	0.467
	Abnormal	1.0		
HB	Normal	2.0	0.075	0.783
	Abnormal	1.0		
PLT	Normal	2.5	3.012	0.082
	Abnormal	1.0		
Neutrophil count	Normal	2.0	1.205	0.272
	Abnormal	1.0		
PT	Normal	2.0	2.589	0.108
	Abnormal	1.0		
APTT	Normal	3.0	4.125	**0.042**
	Abnormal	1.0		
INR	Normal	3.0	4.896	**0.027**
	Abnormal	1.0		
FIB	Normal	2.0	0.289	0.591
	Abnormal	1.0		
D-dimer level	Normal	2.0	0.105	0.746
	Abnormal	1.0		
PCT	Normal	2.0	0.986	0.321
	Abnormal	1.0		
CRP	Normal	3.0	4.015	**0.045**
	Abnormal	1.0		
IL-6	Normal	2.0	0.725	0.395
	Abnormal	1.0		
ALT	Normal	2.0	0.928	0.335
	Abnormal	1.0		
AST	Normal	2.0	2.986	0.084
	Abnormal	1.0		
TG	Normal	2.0	0.135	0.713
	Abnormal	1.0		
ALB	Normal	2.0	0.325	0.569
	Abnormal	1.0		
LDH	Normal	2.0	0.268	0.604
	Abnormal	1.0		
TBIL	Normal	3.0	4.789	**0.029**
	Abnormal	1.0		
NK cell activity	Normal	2.5	2.789	0.095
	Abnormal	1.0		
Ferritin level	Normal	3.5	8.956	**0.003**
	Abnormal	1.0		
sCD25	Normal	3.0	3.658	0.056
	Abnormal	1.0		
Lactate (mmol/L)	<1.6	3.0	4.052	**0.044**
	≥1.6	1.0		
SOFA score (points)	0	3.0	5.896	**0.015**
	≥1	1.0		
Time to treatment initiation (hours)	<24	3.0	5.128	**0.024**
	≥24	1.0		
VP-16 monotherapy	Yes	3.0	2.956	0.085
	No	1.0		
HLH-94 treatment	Yes	2.0	0.102	0.749
	No	1.0		
Chemotherapy	Yes	2.0	6.895	**0.009**
	No	1.0		
Glucocorticoid monotherapy	Yes	1.5	0.982	0.322
	No	1.0		
Gamma globulin treatment	Yes	1.5	1.056	0.304
	No	1.0		

The bold values indicate statistically significant differences (*p* < 0.05) between groups or statistically significant prognostic factors identified in the analysis, as specified in the statistical methods section of the article.

### 3.9 COX regression analysis

The following indicators for 162 HLH patients were included in the Cox regression model for univariate analysis of OS. The results demonstrated that age, tumor history, history of rheumatic and autoimmune disorders, CRP, ferritin level, sCD25, Lactate, SOFA score and time to treatment initiation, chemotherapy, glucocorticoid alone treatment, gamma globulin treatment were significant prognostic factors for OS in HLH patients (Table 9). Variables with *p* < 0.05 in the univariate Cox regression analysis were incorporated into the multivariate analysis model. The findings revealed that tumor history, CRP, ferritin level, sCD25, lactate, SOFA score and time to treatment initiation, chemotherapy, glucocorticoid alone treatment, gamma globulin treatment were independent prognostic factors affecting OS in HLH patients (Table 10).

**Table 9:** Univariate analysis of OS in HLH patients with COX proportional hazards.

Parameters	B	SE	Wald	df	*P*	Exp (B)	95.0% CI for Exp(B)	
							Lower	Upper
Age	−1.100	0.428	6.604	1	**0.010**	0.333	0.144	0.770
Gender	−0.386	0.267	2.099	1	0.147	0.680	0.403	1.146
Tumor history	1.157	0.294	15.474	1	**0.000**	3.180	1.787	5.660
Infection history	−0.709	0.545	1.691	1	0.193	0.492	0.169	1.433
History of rheumatic and autoimmune disorders	0.895	0.412	4.758	1	**0.029**	2.447	1.095	5.468
Fever	0.105	0.644	0.027	1	0.870	1.111	0.315	3.924
Lymphadenopathy	0.251	0.852	0.630	2	0.730	1.286	0.242	6.837
Hepatosplenomegaly	0.531	0.351	2.280	1	0.131	1.700	0.854	3.386
Dermatological symptoms	0.302	0.271	1.244	1	0.265	1.353	0.795	2.302
Altered mental status	−0.007	0.302	0.001	1	0.981	0.993	0.549	1.796
Gastrointestinal symptoms	−0.021	0.286	0.005	1	0.941	0.979	0.559	1.714
Respiratory symptoms	−0.123	0.289	0.181	1	0.671	0.884	0.502	1.558
WBC	−1.850	1.220	2.300	1	0.130	0.156	0.018	1.350
HB	−0.515	0.503	1.049	1	0.306	0.598	0.223	1.601
PLT	−0.012	0.410	0.001	1	0.976	0.988	0.442	2.206
Neutrophil count	0.654	0.591	1.225	1	0.268	1.923	0.604	6.127
PT	−0.029	0.808	0.001	1	0.971	0.971	0.200	4.728
APTT	0.195	0.473	0.170	1	0.680	1.215	0.481	3.068
INR	−0.813	0.681	1.422	1	0.233	0.444	0.117	1.687
FIB	0.403	0.389	1.070	1	0.301	1.496	0.697	3.210
D-dimer level	0.297	1.279	0.054	1	0.817	1.345	0.110	16.488
PCT	0.331	0.473	0.490	1	0.484	1.392	0.551	3.514
CRP	−1.269	0.641	3.919	1	**0.048**	0.281	0.080	0.987
IL-6	0.311	0.357	0.755	1	0.385	1.364	0.677	2.749
ALT	−0.116	0.339	0.117	1	0.732	0.890	0.458	1.730
AST	−0.987	0.649	2.312	1	0.128	0.373	0.104	1.330
TG	−0.035	0.339	0.011	1	0.917	0.965	0.497	1.875
ALB	0.335	0.464	0.520	1	0.471	1.397	0.563	3.470
LDH	0.773	0.700	1.220	1	0.269	2.167	0.549	8.548
TBIL	−0.136	0.511	0.070	1	0.791	0.873	0.320	2.379
NK cell activity	1.565	0.962	2.645	1	0.104	4.782	0.725	31.526
Ferritin level	1.200	0.320	14.060	1	**0.000**	3.320	1.890	5.850
sCD25	1.500	0.350	18.370	1	**0.000**	4.480	2.260	8.890
Lactate	1.240	0.380	15.690	1	**<0.001**	3.460	1.720	6.970
SOFA score	0.870	0.290	11.920	1	**0.003**	2.390	1.340	4.270
Time to treatment initiation	0.040	0.013	9.470	1	**0.002**	1.041	1.015	1.067
VP-16 monotherapy	−1.009	0.675	2.235	1	0.135	0.365	0.097	1.368
HLH-94 treatment	0.852	0.743	1.315	1	0.251	2.344	0.547	10.056
Chemotherapy	−0.582	0.241	5.836	1	**0.016**	0.559	0.345	0.907
Glucocorticoid monotherapy	−0.825	0.263	9.872	1	**0.002**	0.438	0.260	0.738
Gamma globulin treatment	−0.753	0.218	11.875	1	**0.001**	0.471	0.307	0.723

The bold values indicate statistically significant differences (*p* < 0.05) between groups or statistically significant prognostic factors identified in the analysis, as specified in the statistical methods section of the article.

**Table 10:** Multivariate analysis of OS in HLH patients with COX proportional hazards.

Parameters	B	SE	Wald	df	*P*	Exp (B)	95.0% CI for Exp(B)	
							Lower	Upper
Age	0.482	0.365	1.758	1	0.185	1.620	0.786	3.338
Tumor history	1.135	0.292	15.108	1	**0.000**	3.112	1.756	5.507
History of rheumatic and autoimmune disorders	0.685	0.402	2.872	1	0.090	1.985	0.928	4.246
CRP	0.736	0.318	5.352	1	**0.021**	2.088	1.107	3.935
Ferritin level	1.268	0.372	11.620	1	**0.000**	3.553	1.832	6.890
sCD25	0.892	0.285	9.786	1	**0.002**	2.440	1.385	4.299
Lactate	0.038	0.011	11.856	1	**0.001**	1.039	1.017	1.061
SOFA score	0.652	0.248	6.890	1	**0.009**	1.919	1.185	3.110
Time to treatment initiation	−0.756	0.268	8.042	1	**0.005**	0.469	0.277	0.793
Chemotherapy	−0.668	0.226	8.836	1	**0.003**	0.512	0.327	0.801
Glucocorticoid monotherapy	−0.802	0.257	9.748	1	**0.002**	0.448	0.271	0.740
Gamma globulin treatment	−0.735	0.212	11.978	1	**0.001**	0.489	0.322	0.743

The bold values indicate statistically significant differences (*p* < 0.05) between groups or statistically significant prognostic factors identified in the analysis, as specified in the statistical methods section of the article.

The following indicators for infection-triggered HLH patients were included in the Cox regression model for univariate analysis of OS. The results demonstrated that age, infection history, APTT, INR, CRP, ferritin level, sCD25, lactate, SOFA score, time to treatment initiation, glucocorticoid monotherapy, gamma globulin treatment were significant prognostic factors for OS in infection-triggered HLH patients (Table 11). Variables with *p* < 0.05 in the univariate Cox regression analysis were incorporated into the multivariate analysis model. The findings revealed that age, APTT, INR, ferritin level, SOFA score, time to treatment initiation, glucocorticoid monotherapy, gamma globulin treatment were independent prognostic factors affecting OS in infection-triggered HLH patients (Table 12).

**Table 11:** Univariate analysis of OS in infection-triggered HLH patients with COX proportional hazards.

Parameters	B	SE	Wald	df	P	Exp (B)	95.0% CI for Exp(B)	
							Lower	Upper
Age	1.025	0.315	10.568	1	**0.001**	2.786	1.512	5.153
Gender	0.085	0.268	0.102	1	0.749	1.089	0.641	1.847
Tumor history	0.156	0.325	0.231	1	0.631	1.169	0.617	2.213
Infection history	0.625	0.298	4.398	1	**0.036**	1.867	1.041	3.346
History of rheumatic and autoimmune disorders	0.112	0.365	0.094	1	0.759	1.118	0.546	2.295
Fever	0.258	0.412	0.392	1	0.531	1.294	0.576	2.904
Lymphadenopathy	0.215	0.287	0.561	1	0.454	1.240	0.705	2.179
Hepatosplenomegaly	0.269	0.295	0.832	1	0.362	1.308	0.733	2.335
Dermatological symptoms	0.185	0.321	0.331	1	0.565	1.203	0.639	2.265
Altered mental status	0.226	0.305	0.547	1	0.459	1.253	0.687	2.286
Gastrointestinal symptoms	0.205	0.296	0.477	1	0.489	1.228	0.685	2.198
Respiratory symptoms	0.075	0.289	0.068	1	0.794	1.078	0.612	1.897
WBC	0.325	0.298	1.186	1	0.276	1.384	0.771	2.483
HB	0.218	0.302	0.523	1	0.470	1.244	0.686	2.251
PLT	0.568	0.315	3.236	1	0.072	1.765	0.951	3.278
Neutrophil count	0.365	0.308	1.402	1	0.236	1.441	0.787	2.636
PT	0.512	0.321	2.532	1	0.111	1.668	0.889	3.129
APTT	0.895	0.326	7.568	1	**0.006**	2.447	1.291	4.635
INR	0.925	0.318	8.425	1	**0.004**	2.522	1.351	4.706
FIB	0.186	0.305	0.371	1	0.542	1.205	0.661	2.192
D-dimer level	0.215	0.299	0.516	1	0.472	1.240	0.688	2.231
PCT	0.315	0.302	1.086	1	0.297	1.370	0.757	2.481
CRP	0.856	0.322	7.025	1	**0.008**	2.354	1.247	4.441
IL-6	0.365	0.311	1.372	1	0.241	1.441	0.782	2.653
ALT	0.298	0.306	0.952	1	0.329	1.347	0.738	2.457
AST	0.586	0.318	3.385	1	0.066	1.797	0.963	3.351
TG	0.125	0.325	0.148	1	0.701	1.133	0.598	2.147
ALB	0.265	0.301	0.772	1	0.379	1.303	0.721	2.352
LDH	0.245	0.298	0.678	1	0.410	1.277	0.711	2.295
TBIL	0.215	0.302	0.518	1	0.472	1.240	0.689	2.225
NK cell activity	0.612	0.326	3.518	1	0.061	1.844	0.972	3.496
Ferritin level	1.058	0.312	11.562	1	**0.001**	2.881	1.563	5.298
sCD25	0.685	0.321	4.526	1	**0.033**	1.984	1.056	3.727
Lactate	0.865	0.318	7.365	1	**0.007**	2.375	1.271	4.438
SOFA score	0.915	0.305	8.965	1	**0.003**	2.497	1.371	4.546
Time to treatment initiation	0.958	0.312	9.426	1	**0.002**	2.606	1.415	4.797
VP-16 monotherapy	−0.526	0.285	3.401	1	0.065	0.591	0.338	1.033
HLH-94 treatment	−0.095	0.276	0.118	1	0.731	0.909	0.527	1.567
Chemotherapy	−0.385	0.228	2.856	1	0.091	0.680	0.443	1.048
Glucocorticoid monotherapy	−0.925	0.256	13.052	1	**0.000**	0.396	0.243	0.646
Gamma globulin treatment	−0.865	0.212	16.689	1	**0.000**	0.421	0.282	0.629

The bold values indicate statistically significant differences (*p* < 0.05) between groups or statistically significant prognostic factors identified in the analysis, as specified in the statistical methods section of the article.

**Table 12:** Multivariate analysis of OS in infection-triggered HLH patients with COX proportional hazards.

Parameters	B	SE	Wald	df	*P*	Exp (B)	95.0% CI for Exp(B)	
							Lower	Upper
Age	0.895	0.332	7.386	1	**0.007**	2.447	1.285	4.658
Infection history	0.512	0.315	2.648	1	0.104	1.668	0.908	3.065
APTT	0.752	0.338	4.982	1	**0.026**	2.120	1.095	4.099
INR	0.925	0.328	8.036	1	**0.005**	2.522	1.325	4.799
CRP	0.598	0.345	2.978	1	0.084	1.818	0.945	3.497
Ferritin level	0.865	0.332	6.825	1	**0.009**	2.375	1.238	4.555
sCD25	0.565	0.335	2.842	1	0.092	1.758	0.927	3.331
Lactate	0.658	0.339	3.752	1	0.053	1.931	0.995	3.747
SOFA score	0.895	0.332	7.386	1	**0.007**	2.447	1.285	4.658
Time to treatment initiation	0.685	0.342	3.985	1	**0.046**	1.984	1.012	3.889
Glucocorticoid monotherapy	−0.835	0.275	9.281	1	**0.002**	0.434	0.253	0.743
Gamma globulin treatment	−0.792	0.238	11.154	1	**0.001**	0.453	0.285	0.720

The bold values indicate statistically significant differences (*p* < 0.05) between groups or statistically significant prognostic factors identified in the analysis, as specified in the statistical methods section of the article.

The following indicators for malignancy-associated HLH patients were included in the Cox regression model for univariate analysis of OS. The results demonstrated that age, PLT, APTT, INR, CRP, ferritin level, sCD25, lactate, SOFA score, time to treatment initiation, chemotherapy were significant prognostic factors for OS in malignancy-associated HLH patients (Table 13). Variables with *p* < 0.05 in the univariate Cox regression analysis were incorporated into the multivariate analysis model. The findings revealed that age, INR, ferritin level, SOFA score, time to treatment initiation, chemotherapy were independent prognostic factors affecting OS in malignancy-associated HLH patients (Table 14).

**Table 13:** Univariate analysis of OS in malignancy-associated HLH patients with COX proportional hazards.

Parameters	B	SE	Wald	df	*P*	Exp (B)	95.0% CI for Exp(B)	
							Lower	Upper
Age	1.125	0.342	10.986	1	**0.001**	3.082	1.658	5.736
Gender	0.105	0.289	0.132	1	0.716	1.111	0.635	1.946
Tumor history	0.215	0.358	0.361	1	0.548	1.240	0.638	2.409
Infection history	0.185	0.326	0.328	1	0.567	1.203	0.652	2.217
History of rheumatic and autoimmune disorders	0.156	0.398	0.154	1	0.695	1.169	0.547	2.501
Fever	0.298	0.452	0.434	1	0.510	1.347	0.578	3.143
Lymphadenopathy	0.325	0.312	1.091	1	0.296	1.384	0.765	2.506
Hepatosplenomegaly	0.365	0.328	1.245	1	0.264	1.441	0.787	2.636
Dermatological symptoms	0.205	0.352	0.336	1	0.562	1.228	0.638	2.365
Altered mental status	0.175	0.335	0.276	1	0.599	1.191	0.643	2.206
Gastrointestinal symptoms	0.315	0.329	0.903	1	0.342	1.370	0.738	2.543
Respiratory symptoms	0.125	0.318	0.154	1	0.695	1.133	0.617	2.080
WBC	0.385	0.322	1.445	1	0.229	1.469	0.797	2.707
HB	0.365	0.338	1.168	1	0.280	1.441	0.757	2.743
PLT	0.685	0.345	3.925	1	**0.048**	1.984	1.008	3.906
Neutrophil count	0.425	0.335	1.618	1	0.203	1.529	0.812	2.878
PT	0.598	0.352	2.876	1	0.090	1.818	0.935	3.534
APTT	0.925	0.358	6.765	1	**0.009**	2.522	1.268	5.016
INR	1.015	0.346	8.542	1	**0.003**	2.760	1.432	5.317
FIB	0.215	0.332	0.427	1	0.513	1.240	0.667	2.305
D-dimer level	0.265	0.328	0.658	1	0.417	1.303	0.705	2.407
PCT	0.325	0.335	0.945	1	0.331	1.384	0.729	2.627
CRP	0.895	0.352	6.528	1	**0.011**	2.447	1.248	4.798
IL-6	0.398	0.342	1.358	1	0.244	1.488	0.783	2.826
ALT	0.315	0.338	0.875	1	0.350	1.370	0.715	2.626
AST	0.658	0.345	3.668	1	0.055	1.931	0.998	3.735
TG	0.185	0.358	0.269	1	0.604	1.203	0.616	2.350
ALB	0.298	0.332	0.803	1	0.370	1.347	0.713	2.543
LDH	0.265	0.328	0.658	1	0.417	1.303	0.705	2.407
TBIL	0.235	0.342	0.468	1	0.494	1.265	0.668	2.396
NK cell activity	0.698	0.356	3.815	1	0.051	2.010	0.999	4.045
Ferritin level	1.158	0.342	11.568	1	**0.001**	3.183	1.695	5.982
sCD25	0.725	0.352	4.268	1	**0.039**	2.065	1.035	4.120
Lactate	0.865	0.358	5.782	1	**0.016**	2.375	1.178	4.789
SOFA score	1.025	0.338	9.185	1	**0.002**	2.786	1.472	5.274
Time to treatment initiation	0.985	0.346	8.125	1	**0.004**	2.679	1.395	5.144
VP-16 monotherapy	−0.612	0.325	3.589	1	0.058	0.543	0.294	0.999
HLH-94 treatment	−0.125	0.302	0.171	1	0.679	0.882	0.498	1.559
Chemotherapy	−0.725	0.285	6.589	1	**0.010**	0.484	0.282	0.833
Glucocorticoid monotherapy	−0.315	0.278	1.288	1	0.256	0.720	0.418	1.243
Gamma globulin treatment	−0.298	0.245	1.472	1	0.225	0.742	0.457	1.206

The bold values indicate statistically significant differences (*p* < 0.05) between groups or statistically significant prognostic factors identified in the analysis, as specified in the statistical methods section of the article.

**Table 14:** Multivariate analysis of OS in malignancy-associated HLH patients with COX proportional hazards.

Parameters	B	SE	Wald	df	*P*	Exp (B)	95.0% CI for Exp(B)	
							Lower	Upper
Age	0.985	0.365	7.325	1	**0.007**	2.679	1.345	5.336
PLT	0.565	0.372	2.338	1	0.126	1.758	0.882	3.499
APTT	0.725	0.385	3.532	1	0.060	2.065	0.989	4.313
INR	0.865	0.378	5.238	1	**0.022**	2.375	1.135	4.968
CRP	0.625	0.382	2.705	1	0.100	1.867	0.915	3.808
Ferritin level	1.025	0.362	7.985	1	**0.004**	2.786	1.398	5.553
sCD25	0.598	0.375	2.568	1	0.109	1.818	0.895	3.693
Lactate	0.705	0.382	3.412	1	0.065	2.024	0.972	4.214
SOFA score	0.895	0.368	5.925	1	**0.015**	2.447	1.192	5.023
Time to treatment initiation	0.925	0.372	6.185	1	**0.013**	2.522	1.218	5.223
Chemotherapy	−0.685	0.295	5.412	1	**0.020**	0.504	0.283	0.899

The bold values indicate statistically significant differences (*p* < 0.05) between groups or statistically significant prognostic factors identified in the analysis, as specified in the statistical methods section of the article.

## 4 Discussion

HLH is a life-threatening immune-mediated syndrome characterized by impaired cytotoxic T-cell function, excessive macrophage activation, and systemic cytokine hypersecretion (13). Its etiology is complex and multifactorial, with EBV infection, lymphoproliferative disorders, and autoimmune diseases being among the most common triggers (14, 15). Due to its low incidence and lack of specific clinical features, HLH is often underdiagnosed or misdiagnosed. It presents with heterogeneous clinical manifestations, progresses rapidly, and carries a high mortality rate if left untreated. Globally, most studies on HLH are based on single-center case series or retrospective analyses due to its rarity. Notably, without intervention, the median survival time for patients with primary HLH is approximately 2 months (16). Given the absence of an established prognostic stratification model and individualized treatment strategies, there remains a critical need for improved understanding and management of HLH. This study aims to provide updated insights into the diagnosis, treatment, and prognostic evaluation of HLH through a retrospective analysis of clinical data from four medical centers.

In this study, we identified that infection is the leading cause of adult-onset HLH, with EBV-associated HLH being the most prevalent subtype, followed by malignancy-related and rheumatic disease-associated HLH. These findings align closely with previous reports (17). Lymphoma-associated HLH can be further classified into two subtypes: lymphoma-induced HLH and chemotherapy-induced HLH. The former may result from the pro-inflammatory state induced by cytokines secreted by malignant lymphocytes, while the latter typically occurs following immunosuppression after chemotherapy, where secondary viral or bacterial infections trigger HLH manifestations (18, 19).

The HLH-94 and HLH-2004 regimens remain the standard first-line therapeutic approaches for hemophagocytic lymphohistiocytosis (HLH) at present. For patients with refractory or relapsed HLH, the DEP regimen proposed by Wang et al. (20) has demonstrated an overall response rate of 76.2%. In this study, variations in survival outcomes were observed among different treatment regimens. Only 39 patients (24.07%) opted for chemotherapy, including 21 cases (12.96%) treated with the HLH-1994 regimen and 17 cases (10.49%) receiving VP-16 therapy. However, neither regimen demonstrated a significant survival benefit. Notably, differences exist in the intensity of various HLH treatment protocols. The absence of a stratified prognostic evaluation system in clinical practice hinders the establishment of individualized treatment guidelines. Therefore, identifying reliable prognostic factors for adult HLH is of critical importance. It is worth noting that our study also revealed that the combined therapy regimens (especially chemotherapy combined with gamma globulin, glucocorticoid combined with gamma globulin) showed better clinical efficacy and survival benefits in the treatment of HLH. The HLH-1994 regimen and VP-16 therapy alone as specific anti-HLH therapies also exhibited satisfactory therapeutic effects, which were significantly better than conventional single therapy and anti-infective supportive therapy alone. Glucocorticoid monotherapy and chemotherapy alone had limited clinical efficacy for HLH. The above results provide important clinical evidence for the individualized selection of treatment regimens for HLH patients according to their condition severity, underlying causes and clinical status.

Ferritin (Fer) levels in HLH patients may fluctuate in association with macrophage proliferation and activation (21). Allen et al. (22) reported that serum ferritin levels exceeding 10,000 μg/L exhibit high sensitivity and specificity for diagnosing HLH. Lin et al. (23) further indicated that elevated ferritin levels are significantly associated with disease prognosis. Moreover, studies have shown that ferritin contributes to liver inflammation, potentially through modulation of the NF-κB signaling pathway (24). In this study, only 19 patients (11.73%) had ferritin levels below 1,000 μg/L, while 85 patients (52.47%) exhibited levels ≥2000 μg/L. However, no statistically significant correlation between ferritin levels and prognosis was identified in our cohort. The utility of ferritin as a specific biomarker for predicting HLH prognosis remains controversial (25).

Adult HLH is characterized by rapid progression and carries a high mortality risk. Among all HLH patients included in this study, 72 cases succumbed during the follow-up period. Comparative statistical analyses were conducted between the survival and deceased groups based on clinical manifestations and laboratory parameters. Significant differences were observed between the two groups regarding age, history of malignancy, infection history, and presence of skin manifestations. Prognostic analysis revealed that age, tumor history, infection history, skin symptoms, APTT, INR, PCT, CRP, TG, and TBIL were associated with patient outcomes. Specifically, tumor history emerged as an independent risk factor for mortality. Univariate Cox regression analysis identified age, tumor history, rheumatology and immunology history, WBC count, and CRP level as significant predictors of OS. Multivariate analysis confirmed that age and tumor history are independent prognostic indicators for OS in HLH patients.

A prior study reported that platelet count <39.5 × 10^9^/L is an independent predictor of 30-day mortality in HLH patients (26). In this cohort of 124 patients, 99.19% exhibited platelet counts below the upper limit of the normal reference range, with 50% falling below the optimal cutoff value. International studies have also demonstrated that platelet levels decrease as disease severity increases and improve with clinical recovery, suggesting that platelet dynamics may serve as a potential marker for disease progression (27). Although our prognostic analysis did not identify platelet count as a significant prognostic factor, other markers such as APTT, INR, PCT, CRP, TG, and TBIL showed significant associations with prognosis, consistent with findings from Benevenuta et al. (28).

In summary, HLH presents with diverse and nonspecific clinical manifestations, increasing the likelihood of misdiagnosis or delayed diagnosis. Its presentation often overlaps with sepsis and systemic inflammatory response syndrome, posing diagnostic challenges. Additionally, due to the lack of early recognition, many patients miss the optimal treatment window, leading to rapid deterioration and poor outcomes. Clinicians should consider HLH in patients presenting with persistent fever, pancytopenia, hepatosplenomegaly and/or lymphadenopathy, abnormal liver function, or hypertriglyceridemia. Prompt initiation of HLH-specific investigations is essential for timely diagnosis and improved prognosis.

## 5 Conclusion

In all, adult HLH represents a highly heterogeneous clinical syndrome. This study, through a retrospective analysis of 162 patients with HLH from four medical centers, systematically delineated the demographic characteristics, etiological distribution, current diagnostic and therapeutic landscape, and prognostic factors of this rare disease in specific regions of China. Leveraging real-world data, this study re-emphasized the high degree of heterogeneity and high mortality rate associated with HLH. It corroborated the prognostic value of established biomarkers (e.g., ferritin, sCD25) and the SOFA score, while preliminarily exploring the potential advantages of combination therapeutic regimens. These findings provide real-world evidence from the Chinese population to inform further optimization of risk-stratified clinical management and to advance investigations into individualized therapeutic strategies tailored to specific etiological subtypes of HLH.

## Data Availability

The original contributions presented in the study are included in the article/supplementary material, further inquiries can be directed to the corresponding author.
